# Linguistic Analysis of Online Communication About a Novel Persecutory Belief System (Gangstalking): Mixed Methods Study

**DOI:** 10.2196/25722

**Published:** 2021-03-05

**Authors:** Andrew Lustig, Gavin Brookes, Daniel Hunt

**Affiliations:** 1 Department of Psychiatry Faculty of Medicine University of Toronto Toronto, ON Canada; 2 Centre for Addiction and Mental Health Toronto, ON Canada; 3 ESRC Centre for Corpus Approaches to Social Science Department of Linguistics and English Language, Faculty of Arts and Social Sciences Lancaster University Lancaster United Kingdom; 4 School of English Studies Faculty of Arts University of Nottingham Nottingham United Kingdom

**Keywords:** internet, discourse analysis, psychosis, delusions, linguistics, language, online discourse, corpus linguistics, computer mediated communication, schizophrenia, eHealth

## Abstract

**Background:**

Gangstalking is a novel persecutory belief system whereby those affected believe they are being followed, stalked, and harassed by a large number of people, often numbering in the thousands. The harassment is experienced as an accretion of innumerable individually benign acts such as people clearing their throat, muttering under their breath, or giving dirty looks as they pass on the street. Individuals affected by this belief system congregate in online fora to seek support, share experiences, and interact with other like-minded individuals. Such people identify themselves as targeted individuals.

**Objective:**

The objective of the study was to characterize the linguistic and rhetorical practices used by contributors to the gangstalking forum to construct, develop, and contest the gangstalking belief system.

**Methods:**

This mixed methods study employed corpus linguistics, which involves using computational techniques to examine recurring linguistic patterns in large, digitized bodies of authentic language data. Discourse analysis is an approach to text analysis which focuses on the ways in which linguistic choices made by text creators contribute to particular functions and representations. We assembled a 225,000-word corpus of postings on a gangstalking support forum. We analyzed these data using keyword analysis, collocation analysis, and manual examination of concordances to identify discursive and rhetorical practices among self-identified targeted individuals.

**Results:**

The gangstalking forum served as a site of discursive contest between 2 opposing worldviews. One is that gangstalking is a widespread, insidious, and centrally coordinated system of persecution employing community members, figures of authority, and state actors. This was the dominant discourse in the study corpus. The opposing view is a medicalized discourse supporting gangstalking as a form of mental disorder. Contributors used linguistic practices such as presupposition, nominalization, and the use of specialized jargon to construct gangstalking as real and external to the individual affected. Although contributors generally rejected the notion that they were affected by mental disorder, in some instances, they did label others in the forum as impacted/affected by mental illness if their accounts if their accounts were deemed to be too extreme or bizarre. Those affected demonstrated a concern with accumulating evidence to prove their position to incredulous others.

**Conclusions:**

The study found that contributors to the study corpus accomplished a number of tasks. They used linguistic practices to co-construct an internally coherent and systematized persecutory belief system. They advanced a position that gangstalking is real and contested the medicalizing discourse that gangstalking is a form of mental disorder. They supported one another by sharing similar experiences and providing encouragement and advice. Finally, they commiserated over the challenges of proving the existence of gangstalking.

## Introduction

Gangstalking is a novel persecutory belief system whereby those affected believe they are being followed, stalked, and harassed by a large number of people, often numbering in the thousands [1,2]. In contrast to traditional forms of stalking that are usually organized by a single person [3], those affected by gangstalking are unable to identify a single person responsible for their persecution and experience it as a widely distributed and coordinated effort of co-conspirators. People who identify as affected by gangstalking self-identify as targeted individuals.

Although specific experiences of gangstalking vary between those affected, the various expressions of this polythetic belief system include a number of common elements. In particular, the campaign of harassment that affected individuals perceive is frequently experienced as an accretion of innumerable individually benign acts such as people clearing their throat, muttering under their breath, or giving dirty looks as they pass on the street. Perceived as deliberate, connected, and malicious, intense distress is experienced as a cumulative effect of these acts over a prolonged period. Individuals affected by gangstalking are frequently unable to pinpoint a clear motive for the harassment, which is a further source of perplexity and distress. They frequently describe that the apparent goal is to make them appear mentally ill, to cause them to be discredited and disbelieved, and sometimes to encourage or precipitate their eventual suicide.

**Figure 1:**
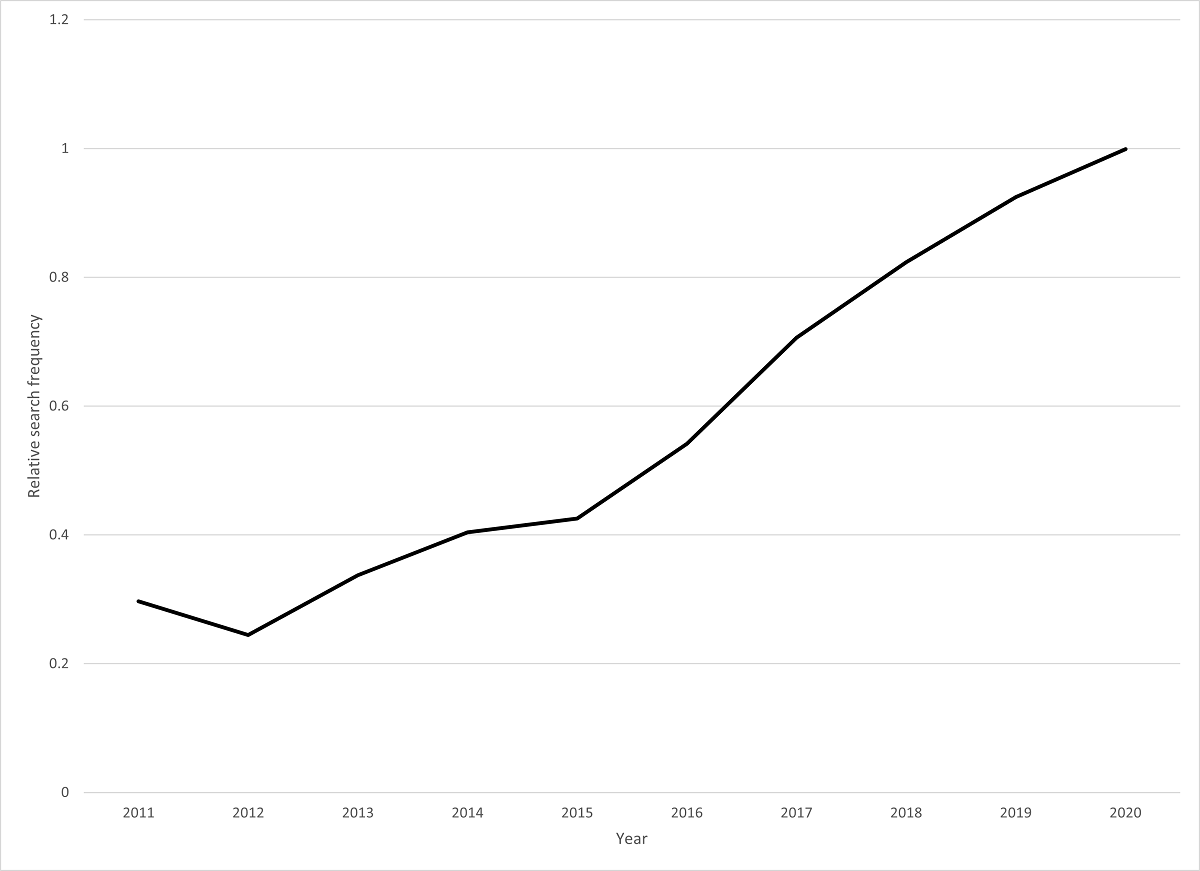
Relative frequency of "gangstalking" as a Google search term.

Interest in gangstalking is increasing over time and the popular press reports the activities of those affected with growing frequency [4-7]. As shown in Figure 1, the popularity of the Google search term *gangstalking* has increased steadily over the past decade [8]. When targeted individuals present to clinical attention, they are frequently diagnosed with psychotic illnesses and the gangstalking is conceptualized as a persecutory delusional system by psychiatric professionals. The gangstalking belief system is similar to some other well-established persecutory delusional belief systems, such as the *Truman Show* delusion [9], where those affected believe that their lives are surreptitiously being continuously recorded and produced into a reality television show and that everyone or nearly everyone they come into contact with is complicit in the deceit. As with many stigmatized beliefs [10,11], individuals affected by gangstalking reject the psychiatric formulation of their condition and turn elsewhere for support.

Targeted individuals congregate in online fora where they can speak openly of their concerns, flesh out their ideas, and comment on each other’s experiences. These fora are a nonclinical environment where those affected may express their beliefs more openly and transparently without the fear of being disbelieved or labeled as may be the case in clinical settings. The internet has become an important source of health information [12]. In addition to providing a platform for those affected to find support, online fora may also serve as a crucible where people flesh out, develop, and linguistically and rhetorically construct the gangstalking phenomenon. It may also serve as a medium of transmission of the ideas as with other belief systems [13]. This study aims to describe how users of an internet forum about gangstalking construct, support, and contest the gangstalking belief system. It also seeks to describe how they use language to navigate social relationships within the context of the forum and as part of these processes.

Delusions are defined as fixed beliefs that are not amenable to change in light of conflicting evidence [14]. An alternative definition is that delusions are beliefs that are demonstrably untrue or not shared by others and which are not ordinarily accepted by other members of the person’s culture or subculture [15]. However, attempts to precisely define delusions have proven problematic and debate and controversy persist [16], with some authors suggesting that pinning down delusions definitively may be an impossible task [17]. For example, superstitious beliefs resemble delusions and are widely held among people who are not affected by mental illness [18]. Other belief systems such as astrology, tarot, and parapsychology also resemble delusional belief systems, yet people endorsing these belief systems are not usually classified as experiencing delusions. Although there are widely accepted hypotheses regarding a biological underpinning of delusions, to date there is insufficient evidence to support a clear mechanistic explanation of them [19]. Moreover, the content of delusions varies across place and time and appears to be heavily influenced by prevalent cultural trends and symbols [20].

For these reasons, we regard persecutory belief systems and their variants such as conspiracy theories, overvalued ideas, and idiosyncratic belief systems as being socially constructed [21,22]. One of the key tenets of social constructionism is that knowledge is sustained by social processes [23]. This view holds that it is through discourse that certain, dominant ways of viewing and understanding particular phenomena come to be regarded as *truth*, at the expense of other perspectives [24]. It is on this basis (ie, through discourse) that certain psychological or embodied experiences come to be understood and treated within a society as being either *normal* or *pathological* and, by extension, those who experience that phenomenon as either *healthy*, *ill*, or even *deviant*. In this paper, we adopt a social constructionist approach to understanding the roles that language, discourse, and other social processes play in constructing the gangstalking phenomenon.

## Methods

The methodology adopted in this study can be described as corpus-based discourse analysis. Corpus linguistics is largely a methodology (but also a field of research) which involves using computational techniques to examine recurring linguistic patterns in large, digitized bodies of authentic language data. Discourse analysis is an approach to text analysis which focuses on the ways in which linguistic choices made by text creators contribute to particular functions and representations. The approach to corpus-based discourse analysis employed in this study is derived from that described at length by Hunt and Brookes [25] in a previous analysis of mental health–related discourse in online fora.

This approach relies on a combination of 3 techniques from corpus linguistics: keyword analysis, collocation analysis, and manual examination of concordances. The first 2 techniques are quantitative methods that use statistical techniques to sift through a large body of text (known as a corpus) to identify, respectively, words and word combinations that are notable due to their high frequency or statistical salience [26]. The third technique, concordancing, is essentially a way of viewing the corpus data that allows users to inspect all instances of a given word, word string, or collocational pairing in the corpus—in context—and, if it is desired, to access the original corpus texts in their entirety. Concordancing facilitates more qualitative analysis of the patterns in a corpus. In this study, it is used to follow up the identification of keywords and collocational pairing, with the ensuing qualitative analysis trained on identifying the wider discursive and rhetorical practices that the keywords and collocates signal and through which the forum users construe their relationships, identity, and experiences.

To obtain source texts for our corpus of forum interactions about gangstalking, we used Google to identify support groups for people experiencing gangstalking. We then focused on the largest gangstalking forum on the internet in terms of number of users, threads, and posts. The forum used to construct the study corpus is organized into topics, each one of which has an accompanying discussion which forms a thread. We used Python 3.0 code to extract 420 complete threads (225,936 words; see Table 1). The data collected included all threads posted between July 17, 2020, and September 2, 2020 (the date of collection). Some threads that were posted and subsequently deleted by their authors were not available for analysis. This was the case for 80 of the 500 threads we sought to extract, which left 420 threads for analysis. The forum requires posters to successfully solve a CAPTCHA before posting to prove they are human and not a bot.

All of the data used in our analysis were posted on a public forum, available to any internet user without having to subscribe or log into the forum. The forum permits users to contribute anonymously with a pseudonymous username that is not linked to their offline identities. Our examination of the forum posts constitutes what Eysenbach and Till [27] refer to as passive analysis. The institutional research ethics board at The Centre for Addiction and Mental Health reviewed the proposed study design and opined that it did not require formal approval.

To help preserve contributors’ anonymity, we term this corpus the *gangstalking internet corpus.* At the time of data collection, the gangstalking forum that we sampled had a total of approximately 14,000 (exactly 13,598) members. To ensure that forum members’ identities are protected as far as is possible, no usernames or references to any other personally identifying information will be reproduced in the data extracts cited in this paper.

Our analysis began by using version 8 of the corpus analytical software *WordSmith Tools* [28] to identify keywords in the study corpus. Keywords are words that occur in the study corpus with a statistically marked frequency when compared with a reference corpus, which usually represents a norm or benchmark for the type of language under study [29]. As our reference corpus, we elected to use the spoken component of the updated British National Corpus [30]—an 11-million-word corpus of conversational British English sampled between 2012 and 2016. This reference corpus was also used by Hunt and Brookes [25], who demonstrated its utility for identifying keywords which signal discursive and rhetorical practices in the context of online fora.

Keyness was measured using a combination of the log-likelihood and the log ratio statistic [31]. Log-likelihood is a confidence measure. The higher the log-likelihood value assigned to a keyword, the smaller the probability that the (statistically marked) observed frequency of that word has arisen due to chance or a sampling error, for example. Log ratio, by contrast, is an effect size measure. The higher the log ratio score assigned to a particular keyword, the larger the observed difference is between its frequencies in the analysis corpus and the reference corpus. We stipulated that keywords should have a log-likelihood score of 15.13, indicating a confidence level of 99.99%. We also specified that a word had to be present in at least 2.5% of forum posts (ie, 69 posts out of 2749) in order to be identified as a keyword. We then ranked the resulting keywords using the log ratio statistic [32]. We set a minimum log ratio of 1.5 for a word to be included as a keyword. A log ratio of 1.5 means that the word is 2.25 times as frequent in the study corpus as in the reference corpus.

After identifying keywords, we grouped them into thematic and semantic categories. We began with the categories defined by Sheridan et al [2] in their content analysis of self-defined gangstalking-affected individuals’ accounts of their subjective experiences of the phenomenon and modified them to capture the themes that emerged from our keyword list.

Following keyword categorization, we extracted collocates of a select number of keywords of interest, in order to examine the wider linguistic contexts within which those words tended to occur in the forum posts. This step takes us beyond the solitary items in the keyword output and begins to move toward understanding the meanings and functions of words in context; as Firth [33] puts it, “you shall know a word by the company it keeps.” In this way, collocate analysis can identify the meanings and associations that affected individuals attribute to different aspects of gangstalking. We defined collocates as words occurring within 5 words to the left or right of the search word (this is the default in *WordSmith Tools* and had been found to be productive for corpus-based discourse studies, eg, by Hunt and Brookes [25]; Tables 2 and 3). Collocation was measured and ranked using the cubed version of the mutual information (MI) statistic (MI^3^). The MI^3^ statistic highlights collocational pairings whose frequency is marked (ie, higher than would be expected given the frequencies of the constituent words and the size of the corpus overall). It is useful for corpus-based discourse analysis, as it favors high-frequency collocational pairings which are thereby particularly well established in the discourse [34]. For analyses of computer-mediated communication, this offers the practical advantage that it does not place undue emphasis on infrequent collocates that are typos or spelling errors.

Finally, keywords and collocational pairings of interest were subjected to manual discourse analysis using concordance output and, where beneficial, based on entire forum posts and those which precede and follow them in the threads. As noted, the objective of this stage of the analysis was to identify the discursive and rhetorical practices through which the forum contributors construed the gangstalking phenomenon and their experiences of it.

**Table 1:** Profile of the gangstalking internet corpus.

Attribute	Gangstalking corpus
Total threads sampled	420
Total posts	2749
Mean posts per thread	6.54
Total words	225,836
Mean words per post	82.1

**Table 2:** Top 5 collocates of gangstalking (5 left/5 right), ranked by MI3^a^.

Rank	Collocate	Frequency	MI^3^
1	*the*	107	16.59
2	*and*	66	14.74
3	*that*	41	13.70
4	*you*	31	12.38
5	*are*	25	12.09

^a^MI^3^: cubed version of the mutual information (MI) statistic.

**Table 3:** Top 5 lexical collocates of gangstalking, ranked my MI3^a^.

Rank	Collocate	Frequency	MI^3^
1	*people*	14	10.32
2	*believe*	12	11.53
3	*think*	12	10.77
4	*One*	11	10.37
5	*Real*	11	12.04

^a^MI^3^: cubed version of the mutual information (MI) statistic.

## Results

### Study Analysis Overview

As described in the previous section, we began our analysis by obtaining keywords from our corpus of gangstalking forum threads. We modified Sheridan and James [1] initial 24 thematic categories of the gangstalking experience to 9 aggregate keyword categories (Table 4): (1) conceptions of gangstalking, (2) social and interpersonal concepts, (3) conceptualizations of the individual, (4) mental and psychological processes, (5) epistemic indicators, (6) extent of conspiracy, (7) technological affordances employed in gangstalking, (8) words pertaining to the internet, and finally, (9) grammatical words were categorized together. Some words were assigned to multiple categories. For example, the polysemous word *state* can refer to a state of mind. It can also refer to a nation or political community. For this reason, it was placed in 2 categories.

**Table 4:** Keyword categories.

Thematic/lexical category	Associated keywords ranked by log ratio score (frequencies [n] in brackets)
Conceptions of gangstalking	*Gangstalking* (380), *gangstalkers* (148), *perps* (113), *gangstalked* (83), *stalkers* (152), *stalked* (99), *stalking* (347), *targeted* (183), *target* (146), *program* (95), *TI*^a^ (144), *evil* (91), *control* (195), *situation* (88)
Social and interpersonal concepts	*Harassment* (161), *victim* (137), *gang* (278), *torture* (141), *other* (152), *power* (111), *involved* (130), *against* (181), *social* (101), *anyone* (225), *help* (223), *group* (127)
Conceptualizations of the individual	*Victim* (137), *individuals* (98), *individual* (104), *human* (110), *life* (345), *person* (275), *someone* (298), *myself* (115)
Mental and psychological processes	*Fear* (120), *mental* (128), *state* (102), *believe* (352), *experience* (121), *crazy* (110), *mind* (276)
Epistemic indicators	*Evidence* (128), *information* (129), *believe* (352), *happening* (94), *real* (190), *reason* (126), *seem* (83), *seems* (100)
Extent of conspiracy	*Government* (200), *public* (120), *police* (176), *state* (102), *law* (115), *using* (130), *world* (240)
Technological affordances	*Technology* (131), *video* (91)
Internet related	*https* (411), *www* (242), *com* (271), *lol* (72), *post* (121)
Grammatical	*etc (167), its* (235), *themselves* (114), *become* (90), *may* (188), *am* (335), *being* (600), *by* (795), *their* (851), *also* (415), *most* (293), *without* (131), *will* (741), *since* (122), *case* (92)

^a^TI: targeted individuals.

### Lexicalizing Gangstalking

The keywords belonging to the category *Conceptions of gangstalking* illustrate that those affected employ various lexical choices for constructing gangstalking in their forum posts. Comparing raw frequencies, it is most commonly referred to as *gangstalking*, which occurs 380 times in the corpus, and *stalking*, which occurs 347 times. *Gangstalking* is the gerund form of *gangstalk*, a portmanteau of *gang* and *stalk*. The word is a neologism. It is not included in standard English language dictionaries and indeed is absent from the updated Spoken British National Corpus, which served as our reference corpus for the keyword analysis above. Gangstalking is sometimes lexicalized as the bigram *gang stalking* in our corpus (n=142 occurrences). The words *stalking* (n=347) and *harassment* (n=161) were also used.

The term *gangstalking* served several different functions in our corpus. In some instances, it serves as a progressive verb. In other instances it is used as a gerund or as a present participle and functions as an adjective. For example:

LIL WAYNE IS **GANGSTALKING** AND HARASSING ME

The **gangstalking** scumbags at the bottom of the hierarchy are usually exploited and disrespected endlessly.

But they still play the childish **gangstalking** games.

While the first of these examples demonstrates that gangstalking is conceived of as a process similar to harassment (and, in this case, perpetrated by a famous musician), examples 2 and 3 demonstrate the way in which the existence of gangstalking is frequently represented as presupposed and uncontroversial. That is, the use of *gangstalking* as a descriptor of people or games functions as an existential presupposition; the use of gangstalking in this way presupposes it is. This implies that gangstalking is a valid and real concept.

The determiner *the* is the most frequent collocate of *gangstalking* in our corpus, occurring in the L1 position (ie, immediately to the left of *gangstalking*) a total of 47 times in the corpus. Lexicalizing *gangstalking* with the definite article *the* frames it entirely as an entity external to the affected individual. Moreover, use of the definite article indicates the verbal, as opposed to the nominal, gerund which conceptualizes a specific and actualized situation that is marked as identifiable [35]. The forum serves as a site of discursive contest between 2 competing worldviews. According to one, the concerns about gangstalking reside within the affected individual as part of a medicalizing discourse. In this paradigm, the experience of gangstalking may be regarded as a chemical imbalance or psychological disturbance. The countervailing view, by contrast, adopts a credulous persecutory discourse and posits that the difficulty is entirely due to the thoughts and behavior of malevolent others located outside of the affected individual. Use of the determiner *the* anticipates this contest and supports the latter view, which is a minority discourse in psychiatric practice, but the majority in this corpus.

Below are several examples of this construction.

Satan is definitely at work when it comes to **the gangstalking** and he is using technology as well as gang stalking perps as human vessels to get his will accomplished.

Even I filed complaints to Federal, provincial, and other organizations, **the gangstalking** increases.

The **gangstalking** was heavy. Every day. Every minute of the day. I still didn’t know what it was. I thought it was bullying, and I “”deserved“” it for being different.

These comments speak about affected individuals’ concern that the phenomenon is widespread, insidious, and centrally coordinated. Much like the use of *gangstalking* as a presupposition, these examples also demonstrate how gangstalking is represented as taking place regardless of the affected individual’s perceptions. This is achieved through the linguistic process of nominalization, in which the process of gangstalking is presented as a noun (the gangstalking). In the second extract above (ie, *Even I filed complaints …*), for instance, it is not that the affected individual *perceives* that the gangstalking is becoming more intense or that they are being gangstalked more frequently, rather their post expresses the seemingly objective fact that their gangstalking has *increased*, once again presenting the phenomenon as incontrovertible.

Representations of the gangstalking phenomenon invariably include references to the perpetrators of the conspiracy as well as affected individuals. In the gangstalking community, the victims are usually known as *targeted individuals*. TI occurred 144 times in the data, *targeted* 183 times, and *individuals* 98 times, while *targeted individual* and *targeted individuals* occurred a total of 86 times. Among those affected, the perpetrators of gangstalking are known as *perps*. That word occurred 113 times in the corpus.

The next most frequent collocate of *gangstalking* is the coordinating conjunction *and*, which occurs most commonly in the R1 position (ie, directly following the node).

I have **gangstalking and** direct energy weapons/remote neural monitoring happening to me.

Can confirm Iran not exempt from **gangstalking and** very advanced mind control technologies.

This **gangstalking, and** chronic chemical poisonings, have taken a toll on my health.

Undoubtedly, these Government stalking worthless punks could not afford nice cars, lavish homes, and domestic fees, if they were not **gangstalking and** research people’s brains 24/7.

As these examples attest, forum contributors use the conjunction to situate the gangstalking behavior within a matrix of similar persecutory and malicious behaviors. In this manner, members of this community construct gangstalking as an individual phenomenon that is intertwined with broader national and international conspiracies. This includes 14 references to “direct energy weapons” and 69 references to voice to skull (V2K) communication technologies. As an online phenomenon, this may also increase contact between individuals who experience gangstalking as an aspect of a persecutory delusion and members of internet conspiracy cultures more generally.

Consistent with this claim, the most frequent keyword in our analysis was *https*, appearing 411 times across 140 of the comments. It was used in the context of URLs, pointing readers to other resources on the internet that commenters used to emphasize and elaborate on their ideas. This speaks to the hyperlinked and connected nature of the internet and online communities but also to the nature of gangstalking as a belief system that has been popularized and shared through the networked communication of the web.

Several of the keywords highlighted interpersonal themes. This demonstrates that the gangstalking belief system is based on malicious interpersonal interactions. Affected individuals identify themselves as *victim* (n=137) and use the term *gang* (n=278) to describe their tormentors. The words *someone* (n=298) and *anyone* (n=225) are used to describe people involved in the conspiracy. Both are indefinite pronouns and allow for doubt about who they are describing. This may speak to the inchoate nature of the belief system in which those affected are certain that they are being targeted even if they cannot always precisely pinpoint whom by.

### Figures of Authority

One of the keyword categories pertains to the broad reach of the conspiracy. These words identify powerful state actors. Almost without exception, those affected cannot identify a single person or agent who is responsible for their persecution. Some affected individuals construe the gangstalking as being retribution for a minor slight or altercation in the past. However, they speak of agents of symbolic authority such as *government* (n=200), *police* (n=176), and *state* (n=102) as either having an organizing role or at least permitting and encouraging the persecution.

Gangstalking definitely is coming from **government**, but it is using the private sector to avoid detection.

The **government** has been using this technology to target specific people and also experimentally torture some people.

Since **police** is involved in this, there is very little we can do about it and sueing them won’t help but don’t let that deter you.

I have a history of being stalked by the **police** so, no, I don’t ask them for help.

This formulation frames gangstalking as a process that is occurring outside the affected individual. However, throughout the corpus, contributors also refer to the alternative view that gangstalking may be a psychological process. The keywords *believe*, *mental*, *mind*, and *experience* all draw attention to the epistemological and ontological challenges faced by those affected: what is really happening and how can one be sure? For example:

Do you actually **believe** in this?

They will not **believe** you. It would be crazy to **believe** us without evidence.

Also, describing gangstalking will often sound completely illogical - people will not **believe** the government would spend that much time or money on a person.

Individuals affected by gangstalking express concern about being able to demonstrate the veracity of their experiences. *Evidence* occurs 128 times in the corpus. It most frequently occurs with the collocates *collect* (MI^3^=15.41) and *gather* (MI^3^=14.18). Those affected post about the need, the challenges, and the potential benefits of accumulating sufficient evidence to conclusively demonstrate the veracity of the belief system:

If you are not presenting some form of **evidence** to skeptics, you are wasting your time.

It sounds like you have the opportunity to gather **evidence** and confirmation this is happening.

Faced with the risk of being disbelieved, being portrayed as mentally ill is a central concern of those affected. A common theme running through their accounts is that the very purpose of the campaign is to discredit and stigmatize them by making them appear chronically mentally ill. For example, the adjective *crazy* occurs 110 times in the corpus with almost all instances pertaining to their concerns about being labeled as mentally ill and stigmatized:

Yeah it’s they ritual to drive you **crazy** so you act weird so they can put you as **crazy** person so nobody will listen to the abuse.

The trick is to be subtle so you don’t come across as **crazy** or threatening.

I was told at the beginning they would make me look **crazy** or lying so no one would believe it.

Throughout the corpus, individuals impacted by gangstalking deal with the possibility and the assertion that they are affected by mental illness. Throughout the corpus, those impacted deal with this tension and the possibility that they are affected by mental illness by representing *craziness* as the intended outcome of gangstalking that they are actively resisting. Accordingly, those affected rarely acknowledge that they have mental illness. However, in some cases posters posit that other forum members do.

You guys are actually insane...

Hey man you need serious mental help.

In other instances, posts note that it is actually the perpetrators of gangstalking that are affected by mental illness:

Most Government gangstalkers dispatched to you, have severe psychological problems, and are afflicted with a serious mental illnesses.

It is not your fault for being gang stalked. Since stalkers have mental illness or personality disorder that fuels this behaviour.

In this manner, references to mental illness in the community serve to insulate the majority of its members from the contention that they themselves are affected by delusions. Mental illness is seen as a characteristic of *perps* rather than targeted individuals or is attributed to a small number of community members whose experiences are dismissed as too extreme.

The frequent use of the word *seem* (n=83) and its variations, *seems* (n=100), *seemed* (n=24), and *seemingly* (n=13), could be viewed as reflecting uncertainty relating to aspects of affected individuals’ accounts of their gangstalking experiences. However, tellingly, these linguistic markers of uncertainty did not reflect any uncertainty relating to the legitimacy of gangstalking itself. Rather, the forum members used *seem* and its related forms to hypothesize about the nature of their own or others’ gangstalking experiences, as well as to theorize about its effects on them as individuals. As the next example demonstrates particularly well, such hypothetical scenarios tend to err on the side of the *gangstalking* explanation for the experiences being described, thereby arguably bolstering the legitimacy of the phenomenon.

...what **seemed** to be the same man, although I couldn’t get a good look at him.

It **seems** like once I feel a great level of peace, they come around to bring me down.

**Seems** like you are being gangstalked by an actual gang.

## Discussion

It is well established that online social support confers mental health benefits upon patients [36,37]. However, the contested nature of gangstalking makes the role of this forum more ambiguous. On the one hand, the forum offers a platform for those affected by gangstalking to be heard and believed, in some instances without the stigma of being labeled as mentally ill. On the other hand, in some instances the forum may serve to further reinforce a maladaptive belief system, drawing those affected further into an echo chamber or down the rabbit hole of conspiracy, reinforcing previously held beliefs and discouraging them from seeking treatment.

Our analysis identified a lexicon comprising words that are highly salient to members of the gangstalking community, many of which are likely to be unfamiliar to outsiders. This includes words like *gangstalking* itself, as well as words that label the various actors in the gangstalking universe including *targeted individuals* and *perp*. In addition, contributors use specialized vocabulary to describe technological affordances such as V2K to describe “voice to skull” technologies to broadcast sounds into the minds of those affected. In addition to its communicative function, using these words serves to validate and legitimize forum contributors as members of the community [38].

The data depict the forum as a site of ontological discursive contest between 2 opposing worldviews about the nature of gangstalking. In one, it is seen as a widespread, crowdsourced system of persecution involving many members of the community, the government, police, and other figures of symbolic authority. The countervailing view is that it is a product of mental disorder and a figment of affected individuals’ imaginations. The linguistic practices in this corpus show that *gangstalking* is lexicalized in various ways that take its existence as given. In addition to constructing and representing coordinated harassment as an objective state of affairs, the nominalization of *gangstalking* also obscures the agent of the harassment and the party affected by it. Contributors use *seem* and its variants to hedge and capture a sense of uncertainty. Further, though gangstalking includes a core set of beliefs [1,2], individual expressions of the belief system vary from person to person. The term *gangstalking* allows forum contributors with varying experiences to have a common nomenclature to refer to their experiences for the purposes of exchanging stories and support with alike others. Moreover, it might be argued that the label *gangstalking* provides the forum users with a means with which to confer a sense of symbolic order over a set of otherwise incoherent experiences, in the process perhaps granting them a sense of control over it [39], or at the very least the linguistic apparatus with which to convey their distress and seek out others who are “in the same boat.”

Despite the potential value of labeling and naming a contested phenomenon like gangstalking, it is nevertheless important to note that many of the contributors to this also manifested a concern about being labeled as mentally ill and generally rejected such a formulation. Although the contributors acknowledge that the distress caused by persecution, alienation, and disbelief may be a source of psychological distress and mental disorder, they also reject the formulation that the belief system is itself a product of the mind. However, some descriptions of gangstalking that are deemed too extreme are labeled as pathological by other group members, which implies that these members operate with a vaguely specified gradient along which experiences of gangstalking may be classed as being pathological at one end and not at all pathological on the other.

Our analysis highlighted the interpersonal nature of the belief system and affected individuals’ concern with interpersonal processes. The gangstalking belief system is characterized by malice perpetrated by a vast number of unnamed others. These include private citizens and also official bodies such as police and government.

These results have the potential to inform clinicians interacting with patients who experience persecutory belief systems. Building a therapeutic relationship to enable engagement is the central process in therapy for psychosis [40]. Having a detailed understanding of the belief systems held by people affected by persecutory belief systems may be important in developing empathy and building a therapeutic alliance. Cognitive behavioral approaches to the treatment of persecutory belief systems recommend that clinicians partner with patients to critically evaluate and dispute delusional and other unhelpful beliefs. Doing so requires a detailed understanding of the beliefs and evidence for and against them. Our hope is that this study may be helpful in that regard.

Our study focused on a particular persecutory belief system. However, insights from this work may be applied more broadly to other, related belief systems. This analysis is particularly valuable because it is based on discussions taking place in a nonclinical setting which arguably allows for more candid and authentic communication, alleviating a potential “Hawthorne effect” of data collected in clinical settings.

Online fora such as the one examined here represent popular avenues for health-related support and advice seeking. This is likely the case, to an extent, for all health-related issues. Yet, this is particularly relevant to contested health issues such as gangstalking, whose contested clinical status may result in those affected turning to peers rather than practitioners for advice and social support. For practitioners seeking to learn about the belief systems and (patient) community norms associated with contested health issues, it therefore behooves them to become acquainted with such online peer support contexts and the linguistic routines (and associated discourses) that characterize the interactions that take place within them.
